# Ethical Considerations about Genomic Medicine Implementation: Lessons Learned from the eMERGE III Study

**DOI:** 10.3390/jpm10040195

**Published:** 2020-10-26

**Authors:** Kentaro Inamura

**Affiliations:** Division of Pathology, The Cancer Institute, Japanese Foundation for Cancer Research, 3-8-31 Ariake, Koto-ku, Tokyo 135-8550, Japan; kentaro.inamura@jfcr.or.jp; Tel.: +81-3-3570-0111 (ext. 5604); Fax: +81-3-3570-0558

**Keywords:** biobank, bioethics, electronic health record, electronic medical record, genetic testing, next-generation sequencing, personalized medicine, precision medicine, research ethics, return of results

## Abstract

The development of high-throughput techniques has permitted the accumulation of enormous amounts of genomic information. As increasing numbers of studies aim to utilize individual genomic information for diagnostic, preventive, or therapeutic purposes, Institutional Review Boards (IRBs) have a greater opportunity to review such types of study protocols. An article published in the *Journal of Personalized Medicine* titled, “Ethical Considerations Related to Return of Results from Genomic Medicine Projects: The eMERGE Network (Phase III) Experience” identified the common concerns of IRBs in the process of reviewing such studies, and some concerns included the readability of informed consent materials, potential risks to participants, information sharing with family members, options for withdrawal or receiving limited results, and provisions to clear participant questions. Since there is an increase in the number of genomic medicine implementation studies worldwide, the insights provided by this study would assist future researchers in protocol preparation as well as aid project review by IRB members.

A huge amount of genomic information is increasingly available due to the advancement of high-throughput techniques (e.g., exome sequencing and genome sequencing). However, numerous barriers have prevented the translation of these data into clinical settings. To identify the optimal methods and practices for the incorporation of genomic information into clinical practice, the National Human Genome Research Institute has sponsored a multicenter consortium called “electronic Medical Records and Genomics” (eMERGE) since 2007 [1]. Currently, the eMERGE (phase III) Network has enrolled approximately 25,000 study participants from 10 clinical sites across the US. The participants, most of whom are healthy and have no medical indication for genomic testing, have undergone next-generation sequencing for 109 actionable disease-related genes and 14 actionable variants. The eMERGE Network has provided a framework for the large-scale clinical translation of genomic data [1]. In an article, published in the *Journal of Personalized Medicine* titled, “Ethical Considerations Related to Return of Results from Genomic Medicine Projects: The eMERGE Network (Phase III) Experience”, Fossey et al. examined the Institutional Review Board (IRB) reviews and decision-making processes involved in the eMERGE III study [2]. The authors successfully provided useful information for investigators and IRB members involved in electronic health record (EHR)-based genomic medicine studies.

According to their study [2], the major issues raised by IRBs while reviewing EHR-based genomic medicine studies were as follows: (I) the readability of informed consent materials, (II) selecting actionable genes in pediatric populations, (III) reporting potential risks to participants, (IV) providing options for withdrawal from the study or receiving limited results, (V) sharing of information with family members, and (VI) establishing the provisions to answer participant questions [2]. (I) IRBs reiterated that consent forms must be easy to understand to ensure that participants can fully comprehend the contents and ask questions. For example, at Mayo Clinic, the IRB recommended explaining the type of genomic screening and sequencing technique in an understandable manner in the consent form. (II) Regarding the process for selecting actionable genes in pediatric populations, it has been stipulated that results for conditions of pediatric onset will be returned to all participants while results for adult-onset conditions will be returned to individuals aged 18 years or older. (III) A number of potential risks of study participation were recognized including the loss of privacy, stigmatization, insurance discrimination, the discovery of non-genetic family relationships, and emotional distress/discomfort. For example, if employers or insurers obtain participant medical information from deposited genomic data, such as evidence of an increased risk of serious or costly disease, they may choose not to employ or insure that participant [3]. (IV) In most studies, IRBs recommended that the participants should be permitted to withdraw from the study at any time before investigators had recorded results in the EHR. However, results could not be removed from a participant’s clinical record once entered in the EHR. At the time of consent, participants in several studies were permitted to choose the types of results they wanted to receive (e.g., primary results, medically actionable results, and all results). A recent study reported that most participants (94.5%) chose to receive all genetic results, whereas the remaining participants selected subsets of results in 10 eMERGE clinical sites [4]. Several sites allowed participants to change their choices at various time points, and 0.5% of participants made changes in their choice of results [4]. (V) Many of the genes tested in the eMERGE study are associated with relatively prevalent autosomal disorders, suggesting the potential need for family (cascade) screening. Some IRBs raised concerns about how participants would be counseled regarding information sharing with family members who might be impacted by the genomic findings. Most studies did not include direct contact with family members in the research protocols. Instead, in an ancillary study at Mayo Clinic, the IRB approved that investigators could contact the first-degree relatives of participants with familial hypercholesterolemia after obtaining participant consent. (VI) IRBs considered it important for study team members to be available to answer any questions that participants may have about the study. They also requested that genetic counseling be readily available to participants with queries about their genomic findings for both positive and negative results. The best method for communicating complex and clinically nuanced findings to participants remains an ongoing challenge in returning genomic test results [5]. Researchers have been recommended to utilize several resources for guidance in this process (e.g., Agency for Healthcare Research and Quality [5,6]).

Studies seeking to implement the findings of genomic analyses are being increasingly conducted globally. Furthermore, comprehensive genomic information is becoming more readily available, and it is being translated to daily clinical settings. Although the study by Fossey et al. was conducted in the US, the underlying principles and study protocol reviews of EHR-based genomic medicine studies are applicable across the world. The authors identified the common concerns of IRBs in the process of reviewing genome medicine implementation studies. The findings would help future investigators and IRB members handle such projects.
